# Bespoke Turing Systems

**DOI:** 10.1007/s11538-021-00870-y

**Published:** 2021-03-19

**Authors:** Thomas E. Woolley, Andrew L. Krause, Eamonn A. Gaffney

**Affiliations:** 1 Cardiff School of Mathematics, Cardiff University, Senghennydd Road, Cardiff, CF24 4AG UK; 2 Mathematical Institute, University of Oxford, Andrew Wiles Building, Radcliffe Observatory Quarter, Woodstock Road, Oxford, OX2 6GG UK

**Keywords:** Turing patterns, Identifiability

## Abstract

Reaction–diffusion systems are an intensively studied form of partial differential equation, frequently used to produce spatially heterogeneous patterned states from homogeneous symmetry breaking via the Turing instability. Although there are many prototypical “Turing systems” available, determining their parameters, functional forms, and general appropriateness for a given application is often difficult. Here, we consider the reverse problem. Namely, suppose we know the parameter region associated with the reaction kinetics in which patterning is required—we present a constructive framework for identifying systems that will exhibit the Turing instability within this region, whilst in addition often allowing selection of desired patterning features, such as spots, or stripes. In particular, we show how to build a system of two populations governed by polynomial morphogen kinetics such that the: patterning parameter domain (in any spatial dimension), morphogen phases (in any spatial dimension), and even type of resulting pattern (in up to two spatial dimensions) can all be determined. Finally, by employing spatial and temporal heterogeneity, we demonstrate that mixed mode patterns (spots, stripes, and complex prepatterns) are also possible, allowing one to build arbitrarily complicated patterning landscapes. Such a framework can be employed pedagogically, or in a variety of contemporary applications in designing synthetic chemical and biological patterning systems. We also discuss the implications that this freedom of design has on using reaction–diffusion systems in biological modelling and suggest that stronger constraints are needed when linking theory and experiment, as many simple patterns can be easily generated given freedom to choose reaction kinetics.

## Introduction

Alan Turing’s chemical theory of morphogenesis Turing (1952) is a remarkable model of spatial pattern formation, providing a mechanistic, predictive framework through which many biological developmental systems can be understood (Woolley et al. 2017; Maini et al. 2012, 2016). Specifically, the theory demonstrates that two distinct diffusible populations (known as morphogens) can produce stationary heterogeneous spatial patterns, if the interactions of the two populations satisfy specific criteria. It has been applied widely to understand biological patterns across numerous scales and taxa (Marcon and Sharpe 2012).

Turing-type patterning is also studied in a variety of other settings, such as in nonlinear optics (Oppo 2009; Ardizzone et al. 2013; Chembo et al. 2017), geochemistry (Baurmann et al. 2004; McBride and Picard 2004), astrophysics (Smolin 1996), reaction–advection–diffusion systems (Klika et al. 2018; Krause et al. 2018a; Van Gorder et al. 2019), network-organised media (Nakao and Mikhailov 2010; Asllani et al. 2014), spatial ecology (Sherratt 2012; Hata et al. 2014; Taylor et al. 2020), and social dynamics (Wakano et al. 2009). Whilst it has been known for decades that chemical systems can be engineered to produce specific Turing patterns (Vanag and Epstein 2001), recently Tan et al. (2018) employed such designed Turing systems to manufacture a porous filter for use in water purification. Experimental efforts are also presently underway to engineer Turing-type patterns in synthetic biological experiments using genetically engineered bacteria (Grant et al. 2016; Boehm et al. 2018; Karig et al. 2018). Alongside a growing theoretical literature on the control of Turing patterns (Pismen 1994; Li and Ji 2004; Kashima et al. 2015), these experimental endeavours demonstrate valuable applications of using engineering design principles to create specific patterns in a variety of settings.

However, despite enormous theoretical and experimental advances, the theory has, so far, had some fundamental obstructions in explaining biological patterns (Woolley et al. 2017). One specific criticism that is often levelled at Turing’s theory is that the parameter region over which patterning can occur is typically relatively small and, thus, fine tuning may become an issue (Murray 1982; Scholes et al. 2019), especially when patterning processes in development occur robustly across a large variety of scales and environments. A number of solutions to this problem have been suggested such as by considering realistic noise in the system, which enlarges the pertinent parameter domain (Woolley et al. 2011; Schumacher et al. 2013; Biancalani et al. 2010), by increasing the number of species that are being modelled (Marcon et al. 2016; Diego et al. 2018), or by considering domain growth, which is ubiquitous in developing systems (Crampin et al. 1999). In contrast to these endeavours, this article provides a constructive user’s guide to building a Turing system that is unstable in a specifically chosen, arbitrarily large parameter region associated with polynomial kinetics (though in principle one can apply this approach to more general self-organisation systems). Consistent with convention, we will call such parameter spaces “Turing spaces”.

This construction highlights several of the inherent difficulties of model selection and parameter identifiability issues that are important across modelling in general, and especially mathematical biology, where universal fundamental principles are rare (Warne et al. 2019; Clermont and Zenker 2015; Maclaren and Nicholson 2019). Specifically, without careful experimental design and rigorous first-principles-based models, the best one can often do is offer a phenomenological interpretation that is consistent with observed data and can be used to hypothesise underlying causal mechanisms. Further experimental work is then required to test any suggested mechanism via theoretically derived predictions. Moreover, the modelling choices that enable consistency of simulation and experiment are frequently motivated by parsimony and Occam’s razor, but this rarely prohibits arbitrarily many possible variations that can consistently match predictions. Of course, phenomenological models can still be useful in providing causal hypotheses, and guiding intuition (Seul and Andelman 1995; Gelfert 2018). However, even if a model is consistent with the underlying mechanisms, it may be impractical, or impossible, to generate data that will specify all parameters uniquely (Anguelova et al. 2007). This paper demonstrates the extreme freedom a modeller has when faced with an observed pattern and a lack of biological constraint within a reaction–diffusion system, whilst highlighting that this freedom results in problems regarding meaningful interpretation. This makes a stronger case for constraining models not only by consistency with particular experimental results but also to known biological pathways and interactions.

**Fig. 1:**
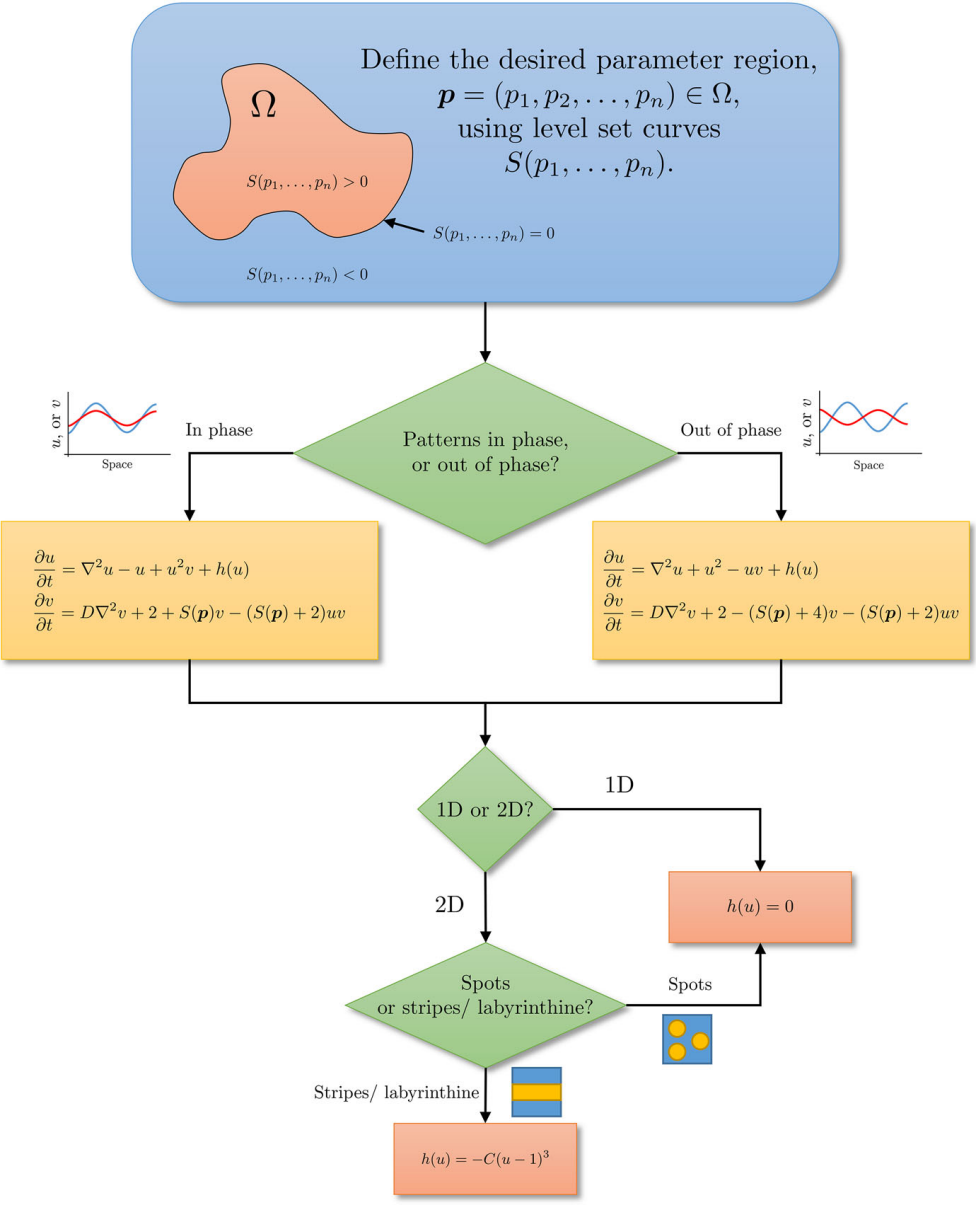
Flowchart for constructing a Turing system that will work in any specified parameter region and provide a desired pattern. Note that *D*, *C*, and the solution domain must be “sufficiently large”. See text for details (Color figure online)

Recent methods have been developed for parameter inference of reaction–diffusion models, subject to a given set of reaction kinetics (Garvie et al. 2010; Krämer et al. 2013; Campillo-Funollet et al. 2019; Kazarnikov and Haario 2020). Such inference methods have been proposed to quantify aspects of signalling dynamics from observed morphogen concentrations in different taxa (Dewar et al. 2010), though again often using phenomenological reaction kinetics that are undoubtedly a caricature of the real morphogen signalling dynamics. Similar ideas have been used to quantify and classify the role of different aspects of agent-based models of pattern formation (such as stochasticity and interactions) in models of zebrafish pigmentation (McGuirl et al. 2020). If the underlying morphogen interactions are known, and hence, the nonlinearities are given, then these techniques provide a powerful way for using observations to determine key features of the morphogen dynamics. However, in practice the choice of the nonlinear kinetics is poorly constrained a priori and one has instead readily observable patterning features. In particular, common classifications of patterns are mainly qualitative. For example, we often seek to specify whether a heterogeneity is that of spots, or stripes, and whether the peaks and troughs of the morphogens are aligned (in-phase patterning) or whether the peaks of one population correspond to troughs of another (out of phase patterning) (see Fig. 1). However, we will show that such qualitative requirements on observable patterns can mean little in constraining the nonlinear kinetics. Hence, a major theme of our results will be advocating for more mechanism-based constraints of the reaction kinetics in reaction–diffusion models of pattern formation.

We will demonstrate that if one has freedom in the choice of reaction kinetics, as is often the case in practice, then model selection and parameter identifiability are theoretically impossible when one simply is considering the presence, or absence, of simple one- and two-dimensional simple patterns (e.g. peaks, troughs, spots, stripes, and labyrinths). In particular, we will demonstrate how to construct polynomial morphogen kinetics such that the patterning parameter domain, morphogen phases, and aspects of the resulting pattern can all be determined at will. We demonstrate examples of these constructions numerically, giving examples of non-contiguous parameter domains where patterning occurs, as well as parameter regimes where the resulting patterns are qualitatively different in specified ways. Such bespoke Turing systems may be valuable for designing chemical, or biological systems, as they will suggest general qualitative features that either have to be (i) discovered, thereby directing future experimental design, or (ii) designed, in the case of synthetic patterning construction. Whilst we will focus on designing a specific chemical system able to match any desired parameter space, the ideas we present should be valuable for the analysis and design of other self-organising systems, and to help illuminate issues of robustness in Turing systems.

Note that the results of this work can either be viewed in a positive or negative light. The positive view commends Turing’s theory for being general enough to mimic a huge range of biological complexity, whilst the negative view suggests that such modelling does not allow us to distinguish Turing’s mechanism from alternative hypotheses without further constraints from biology. This has been discussed widely before, such as by Oster (1988); “So what good are models? The foregoing discussion may seem a bit depressing to theoretical biologists: all manner of models produce the same spatial patterns. Thus it is not generally possible to distinguish between models solely from the patterns they generate, and the question I raised at the beginning begs an answer: what good are models of pattern formation?” Despite some progress, and use of modelling by experimental biologists (Economou and Green 2014), these fundamental issues have not really been overcome. Our work further sheds light on this by demonstrating how easily reaction–diffusion systems can be made to fit arbitrarily complex patterning scenarios. We discuss this further in the conclusion, offering a more nuanced opinion which suggests that this work be viewed as a celebration of Turing’s theory for its beauty, both theoretically and numerically, whilst offering a word of caution as to its over-zealous application to understand any and all pattern formation.

## Framework

In the following, we will consider two continuous populations, *u* and *v*, termed “morphogens”, which is a standard and general name for the populations whose interactions may generate patterning. Critically, we are intrinsically assuming that each population is made up of a large number of individuals, so as to allow a continuum description, although there has been extensive work done on low-copy number reaction–diffusion systems (Adamer et al. 2020; Woolley et al. 2012).

We use the term morphogen to indicate that the populations represent interacting biological/chemical species that are able to generate spatiotemporal complexity. We do not specify the populations’ make up in terms of whether they represent cellular, or chemical populations (for example) (Kondo and Miura 2010). In many cases, these species represent physical, observable quantities and, thus, must be positive to be physically meaningful, though this is not always the case in reaction–diffusion models used for spatial patterning, such as those involving cellular transmembrane potential differences (Sánchez-Garduno et al. 2019). For concreteness, we will require positive solutions throughout, but note that relaxing this requirement makes the analysis below easier, in general.

Our goal is to show that we can construct a set of interaction kinetics, which produce spotted, or striped/labyrinthine, Turing patterns in a specified parameter region, $\Omega \in {\mathbb {R}}^n$. In Sect. 4, we exploit rapid changes in parameter values to demonstrate that we can produce both spots and stripes across different spatial and temporal regions of the same system, effectively sampling from different parameter regimes. Precise details of the resulting patterns will of course depend on geometry and nonlinearity, as well as the heterogeneity employed, but we show that hybrid patterns can be constructed by piecing together different regions in parameter space.

We illustrate a summary of our results in Fig. 1 in the form of a flowchart. We remark that this choice of kinetics, given in the equations in Fig. 1, contains all of the parameter dependencies in the level set function $S(\varvec{p})$, where $\varvec{p}=\left( p_1,\dots ,p_n\right)$ are the parameters, which can be adjusted to construct the desired pattering properties as detailed throughout this paper. Thus, this illustrates how we can design parameter spaces for a desired pattern, rather than be constrained by a specific reaction mechanism.

Furthermore, the basic design principles should be applicable more generally if one has sufficient control over how parameters enter into the system (e.g. through the use of catalysis, reactions on differing timescales, etc.). Additionally, the design framework shown in this toy model has implications for the use of Turing systems in other frameworks as we discuss later.

### Linear Analysis

We first briefly review the basic linear instability analysis for reaction–diffusion systems, in order to motivate our choice of nonlinear kinetics. Let $B\subset {\mathbb {R}}^m$ be a bounded spatial domain with boundary $\partial B$, such that for all $\varvec{x}\in B$, $u(\varvec{x},t)$ and $v(\varvec{x},t)$ are defined to be population densities satisfying the equations:

1 $$\begin{aligned} \frac{\partial {u}}{\partial {t}}&= D_u \nabla ^2 u + f(u,v), \end{aligned}$$

2 $$\begin{aligned} \frac{\partial {v}}{\partial {t}}&= D_v \nabla ^2 v + g(u,v), \end{aligned}$$

where *f*(*u*, *v*) and *g*(*u*, *v*) are functions describing the reaction kinetics. Moreover, $D_u$ and $D_v$ are constant (positive) diffusion coefficients. For simplicity, we assume *u* and *v* satisfy zero-flux boundary conditions on $\varvec{x}\in \partial B$.

In the rest of the paper, we restrict the dimension of *B* to be two, or less, for simplicity of presentation. Our results regarding the existence of Turing patterns and their phase are independent of dimension, whereas the specific results and simulations regarding stripes or spot selection, discussed in Sect. 3.3, are strictly a two-dimensional phenomenon. Although the resulting dynamics can be much richer in more complex cases (for example, through: higher domain dimensions; advective transport; and increased numbers of species (Klika et al. 2012; Marcon et al. 2016; Arcuri and Murray 1986; Aragón et al. 2012; Woolley 2014, 2017; Van Gorder et al. 2019)), the proposed framework can be generalised to these cases. Indeed, with cases of higher complexity there are often even more degrees of freedom to design kinetics that match observed patterns. Here, we consider the simplest case through which we can construct specific kinetic forms of *f* and *g*, in order to satisfy the necessary conditions over a given parameter region for patterns, of any kind, to exist.

For Eqs. (1) and (2) to present a Turing instability, or diffusion-driven instability, the system must satisfy two specific conditions. Firstly, there must be a homogeneous steady state (here taken to be positive), which is stable in the absence of diffusion. Secondly, the steady state must become unstable when diffusion is considered. Hence, we assume that Eqs. (1) and (2) admit at least one positive, constant, homogeneous steady state $(u_c,v_c)$ such that $f(u_c,v_c) = g(u_c,v_c) = 0$. We will neglect pattern formation due to stabilised fronts and other phenomena that can occur in multistable systems (Vastano et al. 1987), focusing solely on patterning due to Turing instability of a single homogeneous steady state. To examine the linear stability of this steady state, we consider a small perturbation,

3 $$\begin{aligned} \left( \begin{array}{c} \hat{u}\\ \hat{v}\\ \end{array}\right) =\left( \begin{array}{c} u_c\\ v_c\\ \end{array}\right) +\left( \begin{array}{c} \epsilon _u(\varvec{x},t)\\ \epsilon _v(\varvec{x},t)\\ \end{array}\right) . \end{aligned}$$

We will consider a one-dimensional interval for the linear derivation, i.e. $B=[0,L]$. Then, the perturbations are assumed to have the following form $(\epsilon _u,\epsilon _v)=(\epsilon _{u0}, \epsilon _{v0})\exp \left( \lambda t\right) \cos (kx)$, where $0<|\epsilon _{u0}|\ll 1$ and $0<|\epsilon _{v0}|\ll 1$ are constants and the cosine form of the perturbation is chosen to satisfy the boundary conditions. We consider a single such Fourier term of wave mode *k* (by considering orthogonality and completeness of such functions). Hence, instability of a particular mode depends on its associated wavenumber, *k*. For this choice of domain, we have that $k=n\pi /L$, for some integer *n*, which is nonnegative, without loss of generality.

Substituting Eq. (3) into Eqs. (1) and (2) and linearising *f* and *g* in the usual way (e.g. see (Murray 2003a)), we can deduce conditions depending on the Jacobian of the kinetics, the diffusion parameters, and the Laplacian spectrum, here given by admissible *k*. The usual criteria for Eqs. (1) and (2) to admit a Turing instability are the following inequalities

4 $$\begin{aligned}&f_u + g_v < 0, \end{aligned}$$

5 $$\begin{aligned}&f_ug_v - f_v g_u > 0, \end{aligned}$$

6 $$\begin{aligned}&D_v f_u + D_u g_v> 2\sqrt{D_u D_v (f_u g_v - f_v g_u)} > 0, \end{aligned}$$

7 $$\begin{aligned}&k^2_{-}< \left( \frac{n\pi }{L}\right) ^2 < k^2_{+} \text { where } \nonumber \\&k^2_{\pm } =\frac{D_vf_u + D_ug_v \pm \sqrt{(D_vf_u + D_ug_v)^2 - 4D_u D_v(f_u g_v - f_v g_u)}}{2D_u D_v}, \end{aligned}$$

where subscripts of *f* and *g* denote partial differentiation with respect to the indexed variable and all partial derivatives are evaluated at the steady state. The first two inequalities (4) and (5) enforce that the steady state is stable in the absence of diffusion. Inequality (6) then ensures that this steady state can be driven to instability when diffusion is included. Finally, inequality (7) states that the patterning domain has to admit an unstable mode and can generally be satisfied for a suitable choice of the integer, *n*, by making the domain sufficiently large, since $k_-<k_+$ if there is a linear instability. Critically, the patterns have an intrinsic wavelength and the domain has to be larger than this wavelength in order for the pattern to appear, at least before nonlinear waveform selection dynamics manifest as patterning develops. We will focus on the first three inequalities (4)–(6), since inequality (7) can always be satisfied by a sufficiently large domain. Whether this size is biologically relevant and/or reasonable depends on the scales involved with the problem and, thus, can only be considered on a case by case basis, in which at least some of the parameters are known to within orders of magnitude.

From considering inequalities (4) and (5), we find that the Turing conditions impose specific sign structures on the partial derivatives (Dillon et al. 1994). Noting $f_u=g_v =0$ is not consistent with relations (4)–(6) and that at least one $f_u$, and $g_v$ must be positive because of inequality (6) then, without loss of generality, we take $f_u>0$, whence the Jacobian of first-order partial derivatives,

8 $$\begin{aligned} \varvec{J}= \left( \begin{array}{cc} f_u &{} f_v\\ g_u&{} g_v \end{array}\right) , \end{aligned}$$

must have one of the following sign structures

9 $$\begin{aligned} \varvec{J}_p= \left( \begin{array}{cc} + &{} -\\ +&{}- \end{array}\right) , \text { or } \varvec{J}_c= \left( \begin{array}{cc} + &{} +\\ -&{}- \end{array}\right) , \end{aligned}$$

and also $$D_v>D_u$$.

Kinetics with the $\varvec{J}_p$ sign structure are known as pure kinetics, and those with the $\varvec{J}_c$ sign structure are known as cross-kinetics. Although, generally, the same patterns are available in each case, the sign structure does influence how these patterns appear in the two morphogen populations, e.g. the peaks and troughs of *u* and *v* will be out of phase in the cross-kinetics case (i.e. peaks of *u* will correspond to troughs of *v*, and vice versa) and in phase in the pure kinetics case, at least for linearised solutions, though, in practice, this relation is typically inherited even when nonlinear dynamics eventually feature.

We will use the sign structure of $\varvec{J}_c$ throughout most of the paper, though note that the following derivation works *mutatis mutandis* in the case that $\varvec{J}_p$ is chosen. We proceed noting that the linear stability of the system is defined completely by the action of the perturbed equations

10 $$\begin{aligned} \frac{\partial {\epsilon _u}}{\partial {t}}&= D_u \frac{\partial ^2{\epsilon _u}}{\partial {x}^2} + f_u\epsilon _u+f_v\epsilon _v, \end{aligned}$$

11 $$\begin{aligned} \frac{\partial {\epsilon _v}}{\partial {t}}&= D_v \frac{\partial ^2{\epsilon _v}}{\partial {x}^2} + g_u\epsilon _u+g_v\epsilon _v, \end{aligned}$$

where $$f_u, f_v>0$$ and $$g_u, g_v<0$$.

We specify new variables

12 $$\begin{aligned} x=[x]x', t=[t]t', \epsilon _u=[\epsilon _u]\epsilon _u', \epsilon _v=[\epsilon _v]\epsilon _v', \end{aligned}$$

where in each case the bracketed term is a constant dimensional scale and the primed term is the new non-dimensionalised variable. Further, we specify the dimensional scales as

13 $$\begin{aligned} {[}t]=\frac{1}{f_u},\quad [x]=\sqrt{\frac{D_u}{f_u}}, \end{aligned}$$

and specify a required equality,

14 $$\begin{aligned} f_u[\epsilon _u]=f_v[\epsilon _v], \end{aligned}$$

which leaves one of the scales, ($[\epsilon _u]$, or $[\epsilon _v]$) as a free parameter. Using this scaling, we have the simpler system

15 $$\begin{aligned}&\frac{\partial {\epsilon _u}}{\partial {t}} = \frac{\partial ^2{\epsilon _u}}{\partial {x}^2} + \epsilon _u+\epsilon _v, \end{aligned}$$

16 $$\begin{aligned}&\frac{\partial {\epsilon _v}}{\partial {t}} = D \frac{\partial ^2{\epsilon _v}}{\partial {x}^2} - F\epsilon _u-G\epsilon _v, \end{aligned}$$

where the primes on the variables have been omitted, and we define $F=|g_u|f_v/f_u^2$, $G=|g_v|/f_u$, and $D=D_v/D_u>1$, with all three parameter groupings strictly positive.

Under this transformation, the Turing instability criteria simplify to

17 $$\begin{aligned}&F>G>1, \end{aligned}$$

18 $$\begin{aligned}&D-G> 2\sqrt{D(F-G)} > 0. \end{aligned}$$

By inequality (17) and the positivity of the parameters, we know that $\sqrt{D(F-G)} > 0$ is guaranteed; thus, we only need to satisfy $D-G > 2\sqrt{D(F-G)}$. Moreover, because $D-G$ grows linearly with increasing *D*, whilst $\sqrt{D(F-G)}$ grows sub-linearly, we are guaranteed to be able to satisfy inequality (18) if we choose *D* large enough. Namely, the minimum possible diffusion is

19 $$\begin{aligned} D_c=\left( \sqrt{F-G} +\sqrt{F}\right) ^2, \end{aligned}$$

which is well defined, because $F>G$ by inequality (17). Although inequality (18) is satisfied for any $D>D_c$, whether *D* (the ratio of the two diffusion coefficients) can be as high as required in a particular application can, once again, only be determined on a case by case basis, through applying the known data to the equations and deriving the appropriate scales.

If we further define $F'=F-1$, $G'=G-1$, then the only criterion that has to be satisfied is $F'>G'>0$. Other than this, we can always choose *D* and *L* large enough to ensure that a system is able to pattern at a suitable wave number. This criterion is not only simple, but also highlights the required relative strengths of the activation and inhibition effects of the two populations. Namely, under the signs of $\varvec{J}_c$ if we identify *u* to be the (self-)activator and *v* to be the (self-)inhibitor, then we see that the strength of inhibition of *u* on *v*, i.e. *F*, has to be stronger than the self-inhibition, i.e. *G*. Equally, $G'>0$ suggests that *G* has to be greater than unity so that self-inhibition is also stronger than self-activation.

Under the signs of $\varvec{J}_p$, although the perturbed system would have the form

20 $$\begin{aligned}&\frac{\partial {\epsilon _u}}{\partial {t}} = \frac{\partial ^2{\epsilon _u}}{\partial {x}^2} + \epsilon _u-\epsilon _v, \end{aligned}$$

21 $$\begin{aligned}&\frac{\partial {\epsilon _v}}{\partial {t}} = D \frac{\partial ^2{\epsilon _v}}{\partial {x}^2} + F\epsilon _u-G\epsilon _v, \end{aligned}$$

we have the same criteria for instability. Once again we see that the activation of *u* on *v* has to be stronger than the self-inhibition of *v*. Thus, in the non-dimensionalised case, where the influence of *u* and *v* on *u* is taken to be the same relative strength, then, regardless of the sign structure, the influence of the self-activator on the self-inhibitor has to be stronger than the self-inhibition. In turn, this influence is stronger than the influence of either morphogen on the activator, which is a useful constraint to place on biological systems that are suggested to act through a Turing instability. Whilst these conclusions are only strictly true in the two morphogen case, analogous results can be drawn for systems with more populations, typically resulting in restrictions to the kinds of interactions between groups of morphogens that can support pattern formation (Satnoianu et al. 2000; Marcon et al. 2016).

We note that all of the parameters from the full nonlinear model that influence linear stability are embedded in the parameters *F* and *G*, corresponding to elements of the Jacobian at the steady state. Suppose our desired parameter domain for a Turing instability, $\Omega \in {\mathbb {R}}^n$, is bounded by the level set curve $S(\varvec{p})=0$, where $\varvec{p}=(p_1,p_2,\dots ,p_n)$ are parameters that influence the reaction kinetics. For orientation purposes let $S(\varvec{p})>0$ for all $\varvec{p}\in \Omega$ and $S(\varvec{p})<0$ otherwise. If we can decompose *S* into the difference of two positive functions, then we can find a system that patterns as required, since $S(\varvec{p}) = F' - G'$ satisfies our constraints. We can choose, for instance,

22 $$\begin{aligned} F'=S(\varvec{p})+\eta , G' =\eta , \end{aligned}$$

for any $\eta >0$, say $\eta =1$. Hence, $F'>G'>0$ if $\varvec{p}\in \Omega$ and, so, we have constructed a linear system that will be Turing unstable in any parameter domain that we choose. Outside of this domain, we have not constrained the system. Thus, patterns may exist, though by construction, the homogeneous steady state will be unstable to spatially constant modes for $S(\varvec{p})<0$. Extending these ideas so that $S(\varvec{p})=0$ gives the boundary of the Turing space, $S(\varvec{p})>0$, whereas the region $S(\varvec{p})<0$ requires a more detailed analysis and is the subject of future work.

### Defining a Full Set of Kinetics

We now specify a set of nonlinear kinetics *f* and *g*. Nonlinearities are needed in the system to bound the otherwise exponential growth of any instability exhibited by the linear system of Eqs. (15) and (16). Further, under our current assumptions, we must also constrain the system to ensure that *f* and *g* never drive the system to negative values of *u* and *v* or suffer from finite time blow up. For simplicity, we consider polynomial kinetics of the following forms:

23 $$\begin{aligned} f=a_1+b_1u+c_1u^\alpha v,\quad g=a_2+b_2v+c_2uv, \end{aligned}$$

where the $\alpha$, $a_i$, $b_i$ and $c_i$ are all constants. To maintain positivity near the origin, we enforce the condition that $a_1$ and $a_2$ are both nonnegative, whilst $b_i$ and $c_i$ are free to take any sign. To constrain the six parameters further, we require a positive steady state. Without loss of generality, we choose (1, 1) to be the critical point; hence, we have to satisfy the equations $f(1,1)=g(1,1)=0$. Further, we require that $f_u(1,1)=1=f_v(1,1)$, $g_u(1,1)=-F$ and $g_v(1,1)=-G$. Solving these equations simultaneously results in the following requirements

24 $$\begin{aligned} a_1 = -2+\alpha ,\quad b_1 = -\alpha +1,\quad c_1 = 1, \quad a_2 = G,\quad b_2 = F-G,\quad c_2 = -F. \end{aligned}$$

Hence, $\alpha$ is the only free parameter and because we require $a_1\ge 0$, then we observe that $\alpha \ge 2$. So for simplicity, we can choose $\alpha =2$. Thus,

25 $$\begin{aligned}&\frac{\partial {u}}{\partial {t}} = \frac{\partial ^2{u}}{\partial {x}^2} - u+u^2v, \end{aligned}$$

26 $$\begin{aligned}&\frac{\partial {v}}{\partial {t}} = D \frac{\partial ^2{v}}{\partial {x}^2} + G'+1+(F'-G')v-(F'+1)uv, \end{aligned}$$

is a pure kinetic Turing system that presents a diffusion-driven instability whenever $F'>G'>0$, given a diffusion constant and domain that are large enough. Consequently, using Eq. (22), with $\eta =1$

27 $$\begin{aligned}&\frac{\partial {u}}{\partial {t}} = \frac{\partial ^2{u}}{\partial {x}^2} - u+u^2v, \end{aligned}$$

28 $$\begin{aligned}&\frac{\partial {v}}{\partial {t}} = D \frac{\partial ^2{v}}{\partial {x}^2} + 2+S(\varvec{p})v-(S(\varvec{p})+2)uv, \end{aligned}$$

presents a diffusion-driven instability whenever $\varvec{p}\in \Omega$.

The results derived for $a_2$ in Eq. (24) demonstrate why we took $\eta =1$, so that $F'=S(\varvec{p})+1$ and $G'=1$. Specifically, if the parameter space defines a closed bounded domain, $\overline{\Omega }=\{\varvec{p}\in {\mathbb {R}}^n: S(\varvec{p})\ge 0\}$, we could have defined $G'=S(\varvec{p})$ and $F'=\sup _{\varvec{p}\in \overline{\Omega }}S(\varvec{p})$, which would also ensure that the Turing instability criterion is only fulfilled when $\varvec{p}\in \Omega$. However, $S(\varvec{p})=G'<0$ outside of $\Omega$ and, so, the positivity of $G'+1$ would not be guaranteed. Consequently, solution trajectories that remain positive for all time (and, thus, physically feasible) are not guaranteed.

Another benefit from defining the desired parameter space using level sets is that since $F'$ and $G'$ are bounded, and able to attain their bounds, we can specify a maximum value for $D_c$. Namely, a Turing pattern is possible for all $\varvec{p}\in \Omega$ if

29 $$\begin{aligned} D>\sup _{\varvec{p}\in \overline{\Omega }}\left( \sqrt{F(\varvec{p})-G(\varvec{p})} +\sqrt{F(\varvec{p})}\right) ^2=\sup _{\varvec{p}\in \overline{\Omega }} \left( \sqrt{S(\varvec{p})}+\sqrt{S(\varvec{p})+2}\right) ^2. \end{aligned}$$

As an even more simple and conservative approach, we could define $D=4\sup _{\varvec{p}\in \overline{\Omega }}F(\varvec{p})=4 \left( 2+\sup _{\varvec{p}\in \overline{\Omega }}S(\varvec{p})\right)$, which always satisfies inequality (29). Further, upon fixing *D* we can calculate the minimum domain, $L_c$, that is required through Eq. (7) and noting that the minimum wave number occurs when $n=1$, namely,

30 $$\begin{aligned} L_c^2>\frac{2\pi ^2 D}{D-G+\sqrt{(D-G)^2+4D(F-G)}}. \end{aligned}$$

We now comment on the $\varvec{J}_p$ sign case as Eqs. (25) and (26), or Eqs. (27) and (28), are only able to produce the $\varvec{J}_c$ sign system. However, the same process as above can be followed except that we use

31 $$\begin{aligned} f=a_1+b_1u^\alpha +c_1u v,\quad g=a_2+b_2v+c_2u v, \end{aligned}$$

and require $f_u(1,1)=1, f_v(1,1)=-1, g_u(1,1)=F, g_v(1,1)=-G$. Consequently, with $\alpha =2$, we are able to create the following pure kinetic system that is unstable whenever $F'>G'>0$

32 $$\begin{aligned}&\frac{\partial {u}}{\partial {t}} = \frac{\partial ^2{u}}{\partial {x}^2} + u^2-uv, \end{aligned}$$

33 $$\begin{aligned}&\frac{\partial {v}}{\partial {t}} = D \frac{\partial ^2{v}}{\partial {x}^2} + G'+1-(F'+G')v-(F'+1)uv, \end{aligned}$$

or whenever $$\varvec{p}\in \Omega $$

34 $$\begin{aligned}&\frac{\partial {u}}{\partial {t}} = \frac{\partial ^2{u}}{\partial {x}^2} + u^2-uv, \end{aligned}$$

35 $$\begin{aligned}&\frac{\partial {v}}{\partial {t}} = D \frac{\partial ^2{v}}{\partial {x}^2} + 2-(S(\varvec{p})+4)v-(S(\varvec{p})+2)uv, \end{aligned}$$

for large enough *L* and large enough *D* (see Eqs. (29) and (30)). Further systems are generated for $\alpha >2$.

Finally, we note that Eqs. (25) and (26) and Eqs. (32) and (33) are highly non-unique. Indeed, we may add any arbitrary terms of the form $(u-1)^\beta (v-1)^\gamma H(u,v)$ where *H* is any smooth function and either $\beta >1,\gamma \ge 0$, or $\gamma >1$ and $\beta \ge 0$. However, we must ensure that these additional terms do not cause the system to present a finite time blow up, which is a weak constraint in practice. In the current case, of considering (*u*, *v*) to be morphogens, we also specify that the choice of *H* should not violate the positivity of the trajectories. However, in more general cases, e.g. one of (*u*, *v*) measuring transmembrane potential differences, this condition can also be relaxed.

Critically, although these extra nonlinear terms will not influence the stability characteristics of the homogeneous steady state at (1,1), they may create new steady states that have different parameter regions of existence and stability. Equally, higher-order terms are able to influence the observed patterns (Ermentrout 1991), which we will see in Sect. 3.3. For simplicity, we are defining our Turing space $\Omega$ in terms of parameters $\varvec{p}$ subject to $D>D_c$, and for *L* sufficiently large. For a bounded patterning space $\Omega$, we can define a largest value of $D_c$ by the supremum over $\varvec{p} \in \Omega$ as in (29). Hence, for all $D>D_c$, $\Omega$ will coincide exactly with the set of kinetic parameters which admit Turing instabilities. This allows us to focus the discussion solely on the kinetic parameters, though in general the domain length scale and ratio of diffusion coefficients should be included in the definition the Turing space. Additionally, if we choose $\Omega$ to be unbounded, then $D_c$ may no longer be a finite number (as one may need larger diffusion ratios to satisfy (18)), and hence, there will still be a dependence of $\Omega$ on *D*. We will now give a few examples of this framework to design parameter spaces, before discussing nonlinear selection effects.

## Examples

Numerical simulations of the following reaction–diffusion systems were run using the finite element software COMSOL Multiphysics 5.3 using a backward differentiation formula scheme in time. The initial conditions are uniformly randomly generated about the steady state 1, with range 0.1. Simulations were run until the supremum of the time derivative over all grid points was less than $10^{-3}$ and until simulation time was greater than or equal to $10^4$. One-dimensional domains were chosen to have a discretisation of at least $10^3$ elements. Two-dimensional domains were chosen to have a discretisation of at least $2.5\times 10^4$ triangular elements. In each case, representative simulations were rerun with halved mesh sizes to ensure that the observed patterns did not change with discretisation.

It should be noted that our stopping tolerances (time greater than $10^4$ and time derivative less than $10^{-3}$) were simply chosen to ensure that the system was close to a steady state. The values themselves have no specific meaning as we are working within a non-dimensionalised setting. However, such thresholds are a critical point for a modeller to consider in applications, as without well-defined scales for the kinetic rates, spatial resolution, and time duration of pattern formation, we cannot conclude, for example, whether $10^4$ time units is a sensible length of time (Woolley et al. 2010).

### Circular Parameter Region

Suppose we want our system to be a pure kinetic system and to pattern within the circular parameter region $\Omega =\{(a,b)\in {\mathbb {R}}|S(a,b)=1-a^2-b^2>0 \}$. Note that we do not make any claims as to the system dynamics outside of $\Omega$. We can define $F'=1-a^2$ and $G'=b^2$ and consider

36 $$\begin{aligned}&\frac{\partial {u}}{\partial {t}} = \nabla ^2 u + u^2-uv, \end{aligned}$$

37 $$\begin{aligned}&\frac{\partial {v}}{\partial {t}} = D\nabla ^2 v + 1+b^2-(1-a^2-b^2)v-(2-a^2)uv. \end{aligned}$$

**Fig. 2:**
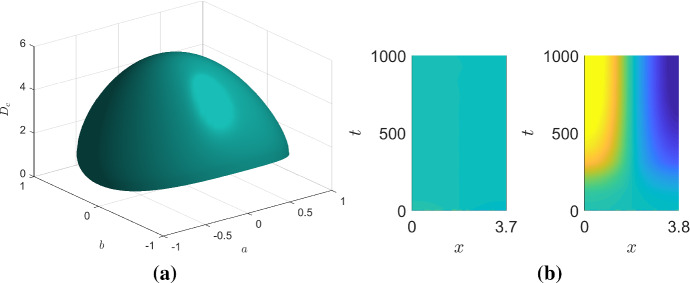
**a** For the case that we require Turing instabilities to occur with the parameter region $\Omega =\{(a,b)\in {\mathbb {R}}|S(a,b)=1-a^2 -b^2>0 \}$ we illustrate the critical values of the diffusion rate, given by Eq. (19), namely $D_c =\left( \sqrt{1-a^2-b^2}+\sqrt{2-a^2}\right) ^2$. The maximum value of $D_c\approx 5.8$ occurs at the origin. **b** Space-time simulations of *u* from solving Eqs. (36) and (37) with $L=3.7$ and $L=3.8$ for the left and right plots, respectively. The simulations demonstrate that although the system may present a Turing instability, the domain has to be large enough for the instability to be realised and the results agree with the theoretical prediction that $L_c\approx 3.77$. Parameters $D=8$, $a=0=b$. The colour axis in both figures runs from blue at $u=0.9$ to yellow at $u=1.1$. Because we have chosen cross-kinetics, the variable *v* (not shown) also presents a single boundary peak that is out of phase with *u* (Color figure online)

By considering the maximum of Eq. (19), plotted in Fig. 2a, we derive that any diffusion rate larger than $D_c=(1+\sqrt{2})^2\approx 5.8$ will cause the system to present a Turing instability for all $(a,b)\in \Omega$. For a conservative approach, we choose $D=\sup _{\overline{\Omega }}4(1+F')=8$. Further, we also need to ensure that the domain size is larger than

38 $$\begin{aligned} L_c= & {} \sup _{\overline{\Omega }} \pi \left( \frac{D}{D - G'-1 +\sqrt{(D-G'-1)^2 - 4D(F' - G')}}\right) ^{1/2}\nonumber \\= & {} \frac{4\pi }{\sqrt{7+\sqrt{17}}}\approx 3.77. \end{aligned}$$

Figure 2b illustrates that even though the bound derived in Eq. (38) is only a sufficient condition, it still serves as quite a sharp bifurcation point, at least in this case. Specifically, for the parameters defined in the caption of Fig. 2b a mode one Turing pattern appears when $L=3.8>L_c$, but not for slightly smaller domains.

All of the analytical results and bounds still hold in two spatial dimensions, with an increase in the number of potential patterning structures. Specifically, whereas in one dimension we are only able to generate peaks and troughs, two dimensions provide the option of stripes, spots and labyrinthine patterns, since there is now a vector of wave modes, $\varvec{k}=(k_x,k_y)$ that can be unstable. Patterning structures become even more complex in three spatial dimensions and beyond (Leppänen et al. 2002, Callahan and Knobloch 1999). Critically, a simple encompassing framework to categorise such higher spatial dimensional patterns does not currently exist, as the distinction relies on nonlinear dynamics (Ermentrout 1991).

**Fig. 3:**
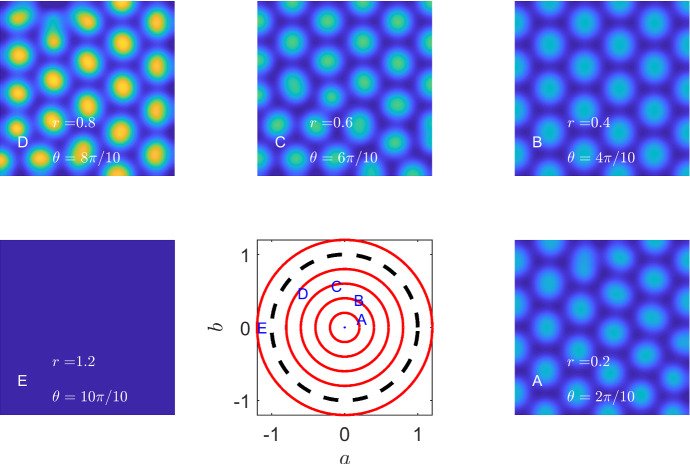
Representative two-dimensional simulations of Eqs. (36) and (37) demonstrating that Turing patterns exist if and only if $(a,b)\in \Omega$, the unit circle, as required. The central image on the bottom row illustrates the parameter region. The red solid lines indicate circles of radii 0.2, 0.4, 0.6, 0.8, and 1.2, respectively, and the black dashed curve represent the circle of radius 1, which is the bifurcation line. The letters **a**–**e** on this subfigure represent the parameter values chosen for each of the surrounding subfigures **a**–**e**. The specific values of radius, *r*, and angle $\theta$ are noted on each subfigure. Each simulation occurs on a square domain of side length 50. The colour axis is the same for all simulations and runs from blue at a value of 0.5 to yellow at a value of 5 (Color figure online)

We now numerically evidence that for the chosen kinetics, all patterns in the circular parameter domain correspond to spot patterns. In order to sweep over $\Omega$, we transform the parameters (*a*, *b*) into their polar forms $a=r\cos (\theta )$, $b=r\sin (\theta )$ and consider multiple values for *r* and $\theta$. Note that simulations were completed over the $(r,\theta )$ domain of $[0,2]\times [0,2\pi )$, using 40 equally spaced values of *r* and 10 equally spaced values of $\theta$. Figure 3 illustrates five representative final steady states of these 400 simulations for different values of $(r,\theta )$. Although pattern alignment and extremal values vary, the patterns within the $\Omega$ parameter region are all spot patterns. By construction, the patterns only exist when $0 \le r<1$ (subfigures A–D) and not for $r>1$ (subfigure E).

### Non-contiguous Parameter Regions

We now give an example of a reaction–diffusion system that is Turing unstable on an unbounded and non-contiguous parameter region, assuming a large enough spatial domain and diffusion ratio, *D*, can be prescribed. To take the complexity one step further, we construct a system that takes a pure kinetic form on one of the regions, whilst taking a cross-kinetic form on the other.

**Fig. 4:**
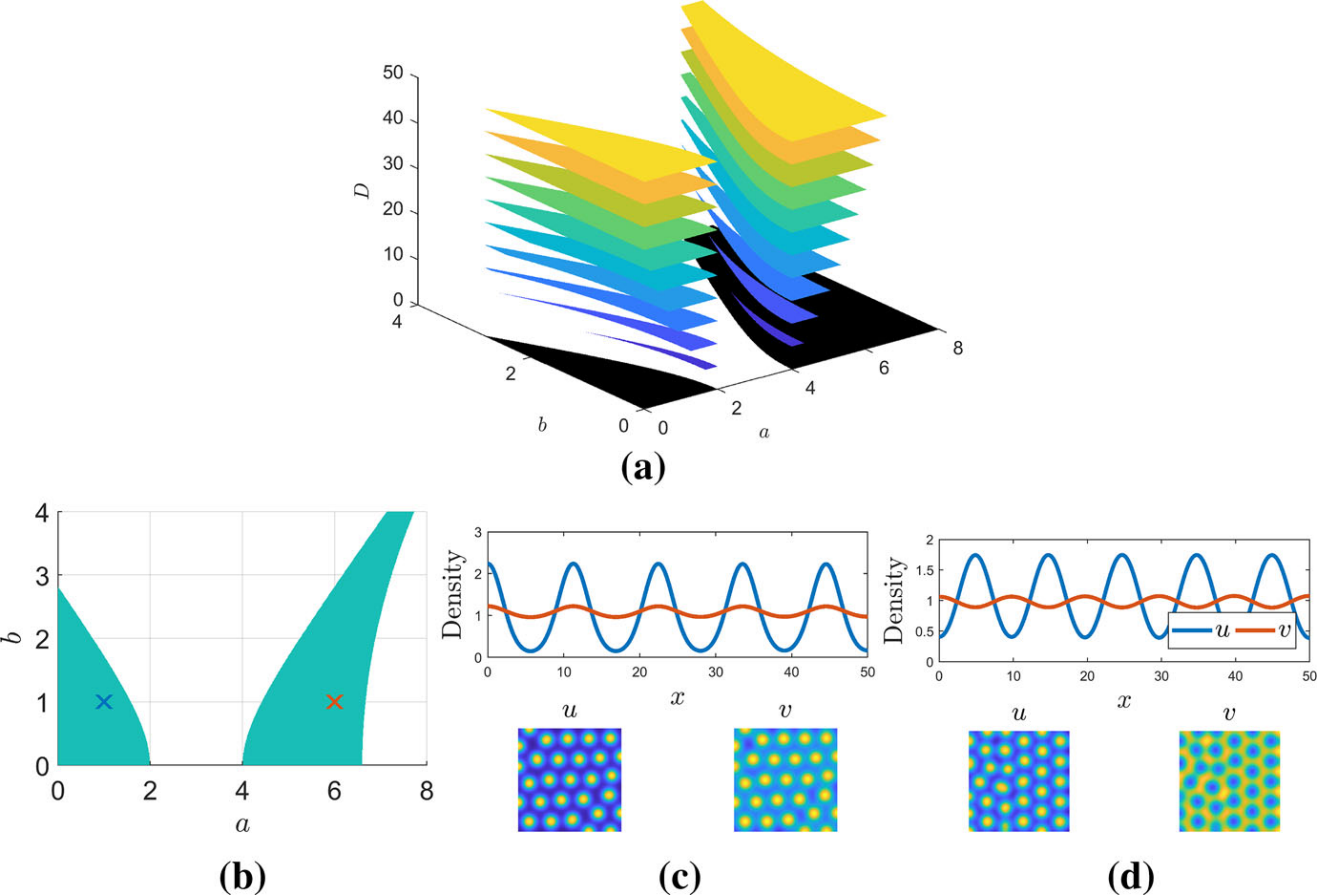
**a** Slices of the (*a*, *b*, *D*) parameter space. The black area is the region $(a-3)^2-b^2\ge 1$ within the range of (*a*, *b*) shown. The coloured slices depict the region of Turing space for given values of *D*. **b** Fixing $D=50$ specifies a suitable non-contiguous parameter space in the (*a*, *b*) parameter plane. The two crosses specify the parameters used for simulations in (**c**) and (**d**), respectively. **c** and **d** are simulations of Eqs. (45) and (46) and illustrate the available patterns in the left and right regions, respectively. The top one-dimensional simulation demonstrates that the pattern is in phase in the left-hand region and out of phase in the right-hand region, as desired. The two-dimensional simulations illustrate both populations, *u* and *v*, demonstrating that the outcome is once again spots, which are either in phase, or out of phase. Parameters $D=50$, **c** $(a,b)=(1,1)$ and **d** $(a,b)=(6,1)$. Each two-dimensional simulation occurs on a square domain of side length 50. The colour axes range from blue at the lowest to yellow at the highest and, from left to right, are: (0.1,3.3), (0.9,1.3), (0.2,2.4) and (0.8,1.1) (Color figure online)

We begin by specifying the disconnected domain,

39 $$\begin{aligned} \Omega =\{(a,b)\in {\mathbb {R}}| a, b>0 \text { and } (a-3)^2-b^2\ge 1\}, \end{aligned}$$

illustrated in Fig. 4a, which specifies the two non-contiguous regions that we will be working with. If we simply wanted to specify that the Turing pattern was active on only both these regions, we could simply use the kinetics derived in Sect. 2.2 using $F=(a-3)^2$ and $G=b^2+1>1$. However, we note that because the space is unbounded, we can no longer specify a single value for $D_c$ for all parameter space. For any given parameter set, $(a,b)\in \Omega$, we are still able to use the inequalities derived in Sect. 2 to create a valid diffusion constant and domain size; however, this has to be computed parametrically. Here, we consider the case that $0\le a\le 6$ and, so, $D=50$ is sufficient (see Fig. 4b).

Critically, further refinement of the kinetics is required to address the constraint that the kinetics are of different types in the different regions. To enforce this, we return to the linearised perturbation system of Eqs. (15) and (16)

40 $$\begin{aligned}&\frac{\partial {\epsilon _u}}{\partial {t}} = \frac{\partial ^2{\epsilon _u}}{\partial {x}^2} + \epsilon _u+\epsilon _v, \end{aligned}$$

41 $$\begin{aligned}&\frac{\partial {\epsilon _v}}{\partial {t}} = D \frac{\partial ^2{\epsilon _v}}{\partial {x}^2} - (a-3)^2\epsilon _u-(b^2+1)\epsilon _v, \end{aligned}$$

and redefine the perturbation $\epsilon _u=\epsilon '_u/(a-3)$; thus, the perturbation changes sign depending on whether *a* is greater than or less than 3. The perturbed system is now of the form

42 $$\begin{aligned}&\frac{\partial {\epsilon '_u}}{\partial {t}} = \frac{\partial ^2{\epsilon '_u}}{\partial {x}^2} + \epsilon '_u+(a-3)\epsilon _v, \end{aligned}$$

43 $$\begin{aligned}&\frac{\partial {\epsilon _v}}{\partial {t}} = D \frac{\partial ^2{\epsilon _v}}{\partial {x}^2} - (a-3)\epsilon '_u-(b^2+1)\epsilon _v, \end{aligned}$$

and, thus, has a cross-kinetic Jacobian sign structure if $a>3$, i.e. (*a*, *b*) is in the right-hand region of $\Omega$, whilst it has a pure kinetic Jacobian sign structure if $a<3$, i.e. (*a*, *b*) is in the left-hand region of $\Omega$. Thus, knowing the structure of the Jacobian that we require, i.e.

44 $$\begin{aligned} \varvec{J}= \left( \begin{array}{cc} 1 &{} a-3\\ a-3 &{} -b^2-1 \end{array}\right) , \end{aligned}$$

we define generic kinetic forms, akin to Eq. (31), and determine the constants in these kinetics analogously to derivations in Sect. 2.2. A suitable set of kinetics is found to be

45 $$\begin{aligned}&\frac{\partial {u}}{\partial {t}} = \nabla ^2 u-u +(4-a)u^2 + (a-3)u^2v, \end{aligned}$$

46 $$\begin{aligned}&\frac{\partial {v}}{\partial {t}} = D\nabla ^2 v +b^2+1+ (3-a)uv+(a-4-b^2)v. \end{aligned}$$

It should be noted here that system (45)–(46) highlights a critical point made earlier that the presented theory guarantees that our patterning requirements are met within the defined parameter region. Outside of this region, we have not provided any constraints. Indeed, in the case of $a=3$ (45) suffers from finite time blow up. However, $a=3$ is outside of the parameter region of interest of $(a-3)^2-b^2\ge 1$. There are many ways of fixing such problems, e.g. by replacing the term $(4-\alpha )u^2$ with $(4-\alpha )u^2/(1+u^2\epsilon ^2)$ for sufficiently small $\epsilon$. However, in this paper we focus on the dynamics within $\Omega$, where $S(\varvec{p})>0$ and leave further subtleties to future work.

Simulations of Eqs. (45) and (46) in the two different regions of $\Omega$ can be seen in Fig. 4c, d. As desired, we observe that, in the both one- and two-dimensional simulations, the constructed equations do, indeed, provide in phase Turing patterns in the left-hand region of $\Omega$ and out of phase patterns in the right-hand region of $\Omega$ (see Fig. 4b).

### Spots to stripes

Thus far, we have demonstrated how to create a set of kinetics that can be tailored to suit any parameter region in either case of wanting the populations in phase or out of phase. Although a greater complexity of pattern is possible in spatial dimensions higher than one, all two-dimensional simulations so far have only demonstrated spot patterns. Hence, in this section we demonstrate that the choice of pattern can similarly be specified as required in the two-dimensional case. Namely, the pattern kinetics can be tuned using a new parameter to provide either spots or labyrinthine patterns depending on the desired application.

Achieving such specificity is an application of a previously proven result, where Ermentrout (1991) used weakly nonlinear perturbation theory to demonstrate that the choice of pattern in a two-dimensional Turing system depends on the nonlinear competition between quadratic and cubic terms in the kinetics. Specifically, if the cubic term dominates, then stripe/labyrinthine patterns are more likely, whereas spots appear in the case that the quadratic term dominates. Critically, the result demonstrated by Ermentrout (1991) assumes a square-based pattern template of spots. This provides the further result that spots and stripes are never stable within the same parameter region. More recent literature (Bozzini et al. 2015; Ma et al. 2019) considers a wider range of stripe and spot arrangements (e.g. rhombi, mixed modes, super-squares, hexagonal, etc.) and demonstrates that although multiple pattern types can be stable at the same time, their existence and stability parameter regions still depend on the interactions of the cubic and quadratic terms. However, it should be noted that domain shape, curvature, and boundary conditions can also influence this bifurcation (Krause et al. 2019, 2020a). Away from boundaries on sufficiently large domains (compared to the pattern wavelength), we anticipate that the results of Ermentrout (1991) hold, as these were derived for periodic square domains and our results demonstrate the anticipated patterning control.

The analysis of the most often observed case, hexagonally arranged spots, is complicated by the fact that its wave mode template produces resonant secular terms in the weakly nonlinear expansion at quadratic order that cannot be completely removed. Rather, a Fredholm solvability criterion has to be applied and assumptions about quadratic terms remaining small are required. Thus, we must remember that all of the reported results only hold in the weakly nonlinear regime and should be taken only as approximate results formally valid only near the boundaries of the Turing regime (where pattern amplitudes are small).

To investigate the influence of cubic terms, we need to extend the basic system (32)–(33). As mentioned in Sect. 2.2, we are able to add any term of the form $(u-1)^\beta (v-1)^\gamma$ with $\beta >1,\gamma \ge 0$ or $\gamma >1,\beta \ge 0$ to Eqs. (32) and (33) and the linear stability analysis stays the same. This suggests that subtracting a term of the form $C(u-1)^3$ from any of the kinetic forms derived here should lead to stripe patterns as *C* is increased, whilst not influencing the desired patterning parameter region. Note that we subtract the cubic term to maintain positivity near $(u=0,v=0)$ and to ensure the cubic term does not risk finite time blow up. Thus, we consider the following equations

47 $$\begin{aligned}&\frac{\partial {u}}{\partial {t}} = \nabla ^2 u-u +u^2v-C(u-1)^3, \end{aligned}$$

48 $$\begin{aligned}&\frac{\partial {v}}{\partial {t}} = D\nabla ^2 v +G'+1+(F'-G')v-(F'+1)uv. \end{aligned}$$

As discussed above, the cubic term does not influence the linear analysis. Thus, patterns are able to form whenever $F'>G'$, assuming *D* and the domain size, $[0,L]\times [0,L]$, are large enough.

Having subjected Eqs. (47) and (48) to a weakly nonlinear expansion technique (not shown), it appears that the dynamics we are interested in (namely the ability of the *C* parameter to control pattern type) is outside the scope of the weakly nonlinear regime. Specifically, although for small *C* we are able to track the start of bifurcation from the stable homogeneous state to stable heterogeneous patterns, the point at which the patterns cease to be stable is defined at a higher order. Further, as we will see in the upcoming simulations, as *C* increases the spot pattern begins to bifurcate from the stripe solution, rather than from homogeneity, a result that is completely outside weakly nonlinear analysis. Thus, to understand the bifurcation properties of system (47)–(48) in more detail, we turn to simulation. Namely, we use the MATLAB numerical continuation software package pde2path, which is designed to derive PDE bifurcation structures (Uecker et al. 2014; Dohnal et al. 2014; Engelnkemper et al. 2019).

**Fig. 5:**
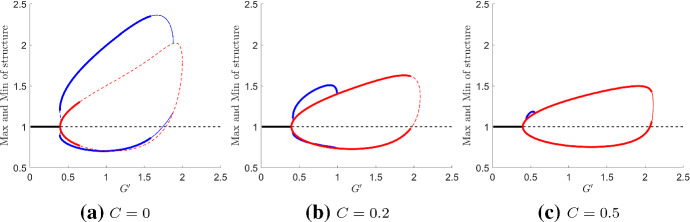
Bifurcation diagram of the steady states of Eqs. (47) and (48) on a square domain of side length 10 presenting the maximum and minimum of the patterns in the *u* population. The lines indicate the parameter dependence of the homogeneous solution (black), stripe solution (red), and hexagonal spot solution (blue). The thick solid lines represent the Max and Min values of stable solutions, whilst the thin dashed lines represent the Max and Min values of unstable solutions, and the graphs illustrate that stripe solutions dominate at larger *C*. Parameters: $D=9$, $F'=2$ and *C* are shown below each figure

Figure 5 illustrates that the numerical continuation matches the expected results from the literature (Ermentrout 1991; Bozzini et al. 2015; Ma et al. 2019), namely that as the cubic term increases, the system tends to present labyrinthine patterns. Specifically, in Fig. 5a, we observe that the spot solution branch bifurcates subcritically and as *C* increases, the region of stable spot pattern existence shrinks. Further, although stripe patterns are available when $C=0$, we see that their region of stability increases dramatically as *C* increases (compare the red curves in Fig. 5a, b). Finally, by the time $C=0.5$ the spot solution barely exists (see Fig. 5c).

Using these ideas, we now extend the non-contiguous parameter region kinetics, Eqs. (45) and (46), to possess either stripe or spot patterns depending on parameter choices. Taking the system from Sect. 3.2, we subtract a cubic term,

49 $$\begin{aligned}&\frac{\partial {u}}{\partial {t}} = \nabla ^2 u-u +(4-a)u^2 + (a-3)u^2v-C(u-1)^3, \end{aligned}$$

50 $$\begin{aligned}&\frac{\partial {v}}{\partial {t}} = D\nabla ^2 v +b^2+1+ (3-a)uv+(a-4-b^2)v. \end{aligned}$$

**Fig. 6:**
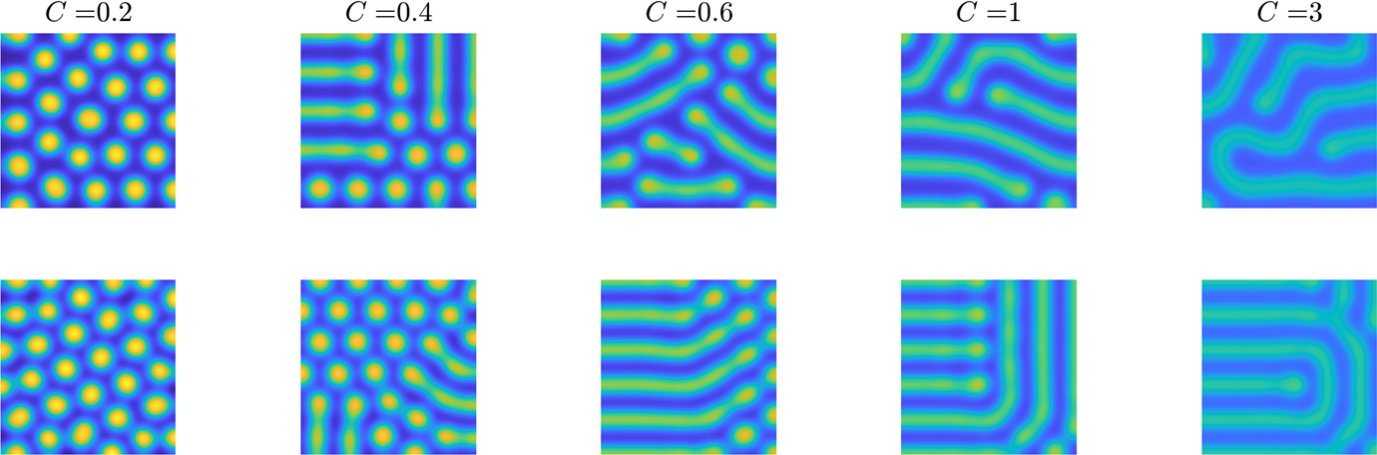
The action of increasing the influence of a cubic term in Eqs. (45) and (46). Each two-dimensional simulation illustrates *u* on a square domain of side length 50. The colour axes range from blue at the lowest to yellow at the highest, and the top row is plotted on a consistent range of (0.2, 2.6), whilst the bottom row is plotted on a consistent range of (0.4, 2). The top row parameters are $(a=1, b=1)$, and thus, *v* will be in phase. The bottom row parameters are $(a=6, b=1)$, and thus, *v* will be out of phase

Figure 6 illustrates the influence of increasing *C* on the pattern structure for both pure (top row) and cross (bottom row) kinetics. As expected, we observe in both kinetic types that as the value of *C* increases left to right, the pattern transitions from spots to labyrinthine structures. We also note the amplitude of the pattern is suppressed with increasing *C* due to the cubic term having an inhibitory role.

## Applications Beyond Homogeneous Systems

**Fig. 7:**
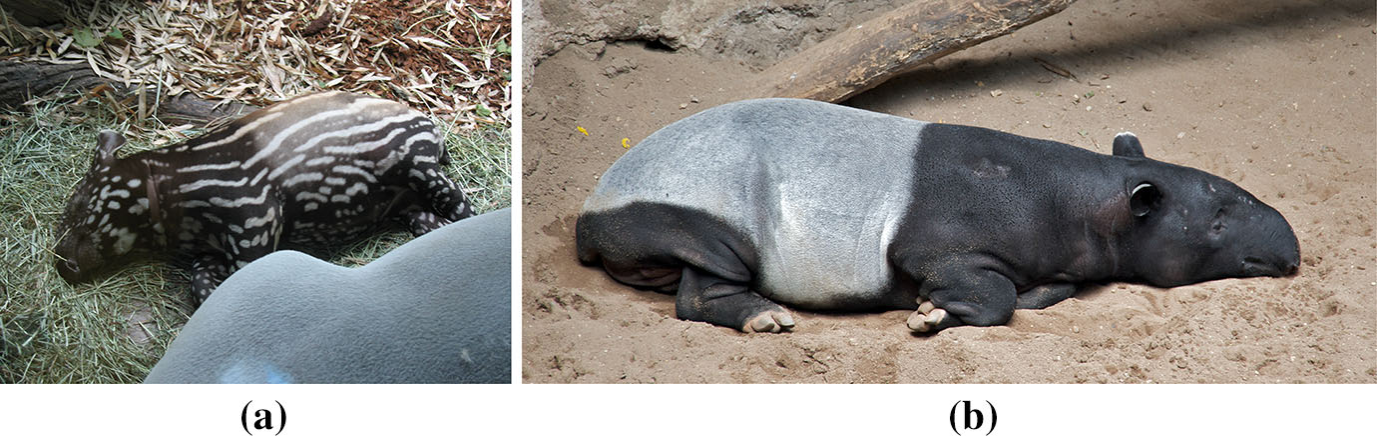
Pattern evolution on a Malayan Tapir going from **a** broken stripes and spots on the baby to **b** large banded colouring on the adult. Any patterning theory for such an animal would have to account for this spatiotemporal complexity

As a final example, we extend the model given by Eqs. (49) and (50) to have spatial and temporal heterogeneity, to obtain an enormous range of patterns. Using such heterogeneities, one can in principle design quite elaborate patterning fields that can change across space and time. It should be noted that including such spatiotemporal heterogeneities is not simply a matter of mathematical interest, as many biological systems demonstrate such complexity (Maini and Woolley 2019). For example, Malayan tapirs are born with dappled white stripes and spots on a brown coat. Critically, these patterns do not follow Turing’s simple theory relating spatial size and pattern complexity, namely, thinner/ smaller domains should have simpler patterns than larger domains, whereas tapirs have stripes on their bodies breaking up to spots on its legs. Moreover, these patterns disappear during the first few months after birth and transition into a large-scale striped pattern of dark head and front legs, light body, and dark hind legs (Othmer et al. 2009) (see Fig. 7). Furthermore, transitions between solid spots and “rosette” patterns can be observed in leopards and jaguars as they mature (Liu et al. 2006; Werdelin and Olsson 1997). We do not claim that these evolving patterns are necessarily due to reaction and diffusion, only that there is an interest in extending such pattern forming systems to include spatial and temporal complexity.

There are many technical difficulties in generalising the linear theory explored in earlier sections, though some progress has been made; see, for instance, Krause et al. (2020b), Kozák et al. (2019) for spatial heterogeneity and Klika and Gaffney (2017), Madzvamuse et al. (2010), Van Gorder (2020) for temporal forcing. We also remark that a large number of papers have explored these concepts, especially in terms of precursor patterns or environmental modulation as cited within the preceding references. Broadly summarising this earlier published work, whilst such heterogeneity can induce novel effects, if it is either sharply separated, or accompanied by sufficiently small diffusion, then the stability and instability criteria of a linear analysis hold locally, with only minor generalisation in the analogous result for slow temporal forcing (Madzvamuse et al. 2010). Thus, we anticipate the validity of linear stability and instability criteria applied locally for the stripe and spot transitions, considered here.

We focus on demonstrating our results through simulation, leaving a detailed mathematical justification for future work. Whilst our intuition for the “localization” of patterning above can be justified near a steady state, we will show that the predictions of the linear theory, developed in the papers cited above, appear to hold even when transitioning between patterned states which are far from equilibrium. Justifying such evolving states is beyond the scope of what we aim to do here, which is merely to demonstrate the ability of a modeller to design sophisticated patterns in space and time.

To proceed, we replace *C* and *a* with functions of space and time, which we will specify for our examples. We also assume that the linear and weakly nonlinear results of the previous sections hold, at least for well-chosen functions, and give two examples where spatial and temporal heterogeneity interact with the patterning process in interesting ways. In both examples, we will consider a domain of size $120\times 60$ where half of the domain is in a stripe parameter regime, and the other half in a spot regime. We first demonstrate switching between these in time by changing the local parameters in each to oscillate between stripe and spot parameters, all by a simple choice of the function *C*. In the second example, we leave both spatial regions fixed, but instead exploit the parameter *a* to globally change between pure and cross-kinetic systems, so that *u* and *v* change phase.

For the first example, we set $a=1$ and

51 $$\begin{aligned} C = 1-H\left( \cos \left( \frac{2 \pi t}{6000}\right) \right) +\left( 2H\left( \cos \left( \frac{2 \pi t}{6000}\right) \right) -1\right) \left( \frac{1+\tanh (x-60) }{2}\right) , \end{aligned}$$

where *H* is a sharp Heaviside step function. Hence, *C* rapidly transitions between $C=0$ and $C=1$, both in time and across the domain, giving a spatiotemporal transition from spots to stripes as shown for constant *C* in Fig. 6. The spatial heterogeneity is sharp across the midpoint of the domain (though it is smoothed slightly to avoid numerical errors in the finite element discretization across this interface). The temporal switching changes the kinetic parameter at this interface every $t=3000$ units of time, which is sufficient time for patterns to get close to an equilibrium state. As a technical aside, we note that we increased the number of triangular finite elements for these simulations to $\sim 50,000$ and restricted the maximum timestep of the solver to 10 in order to resolve the temporal switching.

**Fig. 8:**
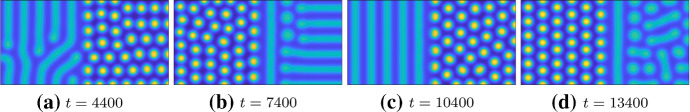
Simulations of Eqs. (49) and (50) with Eq. (51) on a domain of size $120 \times 60$ with $a=1$, $b=1$, and $D=50$ at different points in time, specified under each figure. The plots show simulations of *u*. The *v* pattern (not shown) is in phase and is, thus, qualitatively similar to *u*. The times correspond to 100 time units before the next transition. *u* ranges from 0.08 to 3.3

We plot example simulations of this system in Fig. 8, demonstrating fully formed patterns across the domain, which oscillate in space and time between stripes and spots. We note that the “stripe” parameter regime does not always give labyrinthine structures as it sometimes degenerates into spots, but the maximal value of *C* can be increased such that this does not happen. (Multiplying the $\tanh$ in (51) by 5, for instance, is sufficient to do this, but at the cost of making the amplitude of the stripes lower and hence harder to see against higher-amplitude spots.) We note that whilst the linear analysis is not valid here, as we are really transitioning from a completely patterned stated to another with the temporal oscillation, the emergent bifurcation structures predicted by the linear and weakly nonlinear theories seem to be captured in these simulations.

We next give an example of changing the parameter *a* in order to oscillate between in and out of phase kinetics in time, again on a spatial domain, which itself is composed of stripe and spot regions. We take,

52 $$\begin{aligned} C = 1+\tanh (x-60), \quad a = 1+5H\left( \cos \left( \frac{2 \pi t}{6000}\right) \right) , \end{aligned}$$

with *H* again being the Heaviside, and the same considerations as above regarding smoothed spatial steps and sharp temporal ones. Here, *C* transitions from 0 to 2 over the spatial domain, whilst *a* oscillates in time from 1 to 6, as in the static examples given in Fig. 6. As before, we use the same slow period of oscillation so that the kinetics stay approximately constant in time for 3000 units of time.

**Fig. 9:**
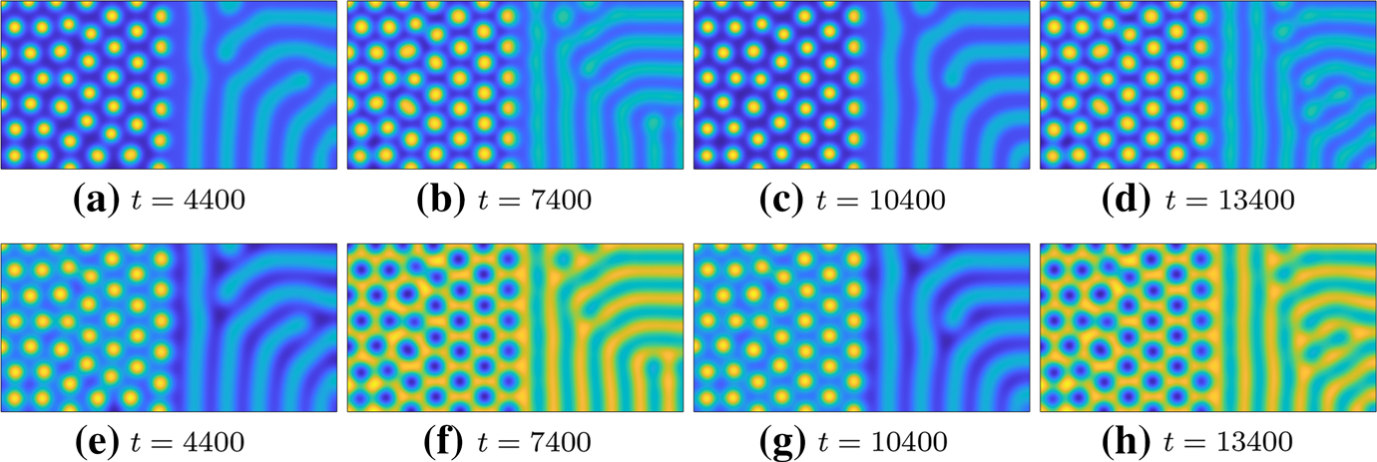
Simulations of Eqs. (49) and (50) with Eq. (52) on a domain of size $120 \times 60$ with $b=1$, and $D=50$ at different points in time. The top row shows simulations of *u*, and the bottom row simulations of *v* at the given times, which correspond to 100 time units before the next transition. *u* (top) ranges from 0.08 to 3.3, and *v* (bottom) ranges from 0.9 to 1.3

Snapshots of simulating Eqs. (49) and (50) with parameters (52) are illustrated in Fig. 9, where we observe that even though both *u* and *v* are driven by temporally evolving kinetics, it is primarily *v* that toggles its phase. Further, the pattern structures remain broadly intact (even when carried out for several more oscillations), though we do see some changes in the striped half of the domain, as these structures are generally less stable to perturbation than spots. Simulations of only spot dynamics (not shown) confirm this as we can indefinitely oscillate between in and out of phase kinetics without modifying the number or organization of spot patterns, as long as sufficient time is given for the pattern to relax between such oscillations.

### Patterns on Complex Prepatterns

Thus far, we have only demonstrated simple binary heterogeneities in space and time. In real biological systems, complex heterogeneity is more often the rule, with these simple ideal cases the exception (Weber et al. 2019; Warmflash et al. 2014; Woolley et al. 2014). If the patterning wavelength is sufficiently smaller than the length scale of any background or exogenous heterogeneity, then these observations about localised patterns can be shown more formally for sufficiently small diffusion, regardless of the diffusion ratio, *D*, and such scales often apply in development, for instance (Krause et al. 2020b). Hence, even extremely complicated underlying prepatterning systems can often lead to simple combinations of spot and stripe structures, and one can use this intuition to engineer hybrid patterns in a piecewise fashion.

**Fig. 10:**
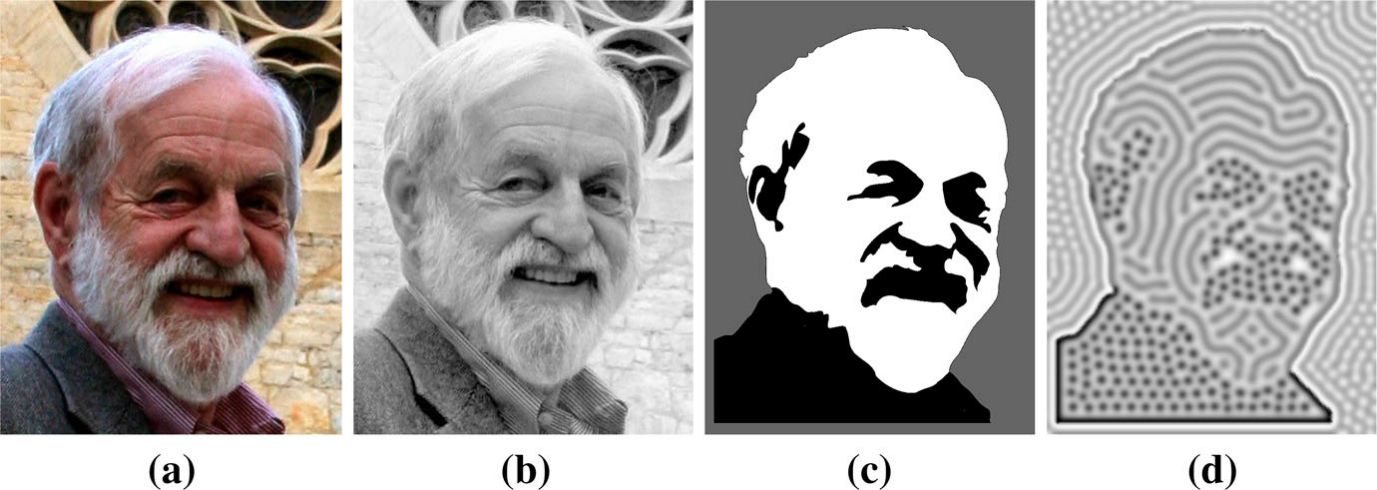
Imaging pipeline from initial photograph to final Turing pattern with a complex heterogeneity. **a** Initial photograph. **b** Photograph has been converted to greyscale. The photograph also has enhanced contrast to emphasise the different regions. **c** Segmented image using intensity as a segmentation parameter. This is used as a parameter prepattern for Eqs. (49) and (50). Namely, $C=1$ and $a=1$ within the black region, $C=0$ and $a=1$ in the white region and $C=0.4$ and $a=6$ in the grey region. In terms of the Turing pattern in the concentration of *v* in **d**, this provides regions of: spots in the black regions; stripes in the white region and inverse spots and stripes in the grey region. Other parameter values: $D=50$, $a=1$ and the patterning domain size is equal to the pixels in the photograph, namely $(0, 558)\times (0, 734)$. The colour axis goes from 0.8 (light) to 1.5 (dark)

As a first demonstration, we construct a system that presents distinct regions of patterning behaviour due to a complex heterogeneity. The heterogeneity is taken from a photograph of James Murray, author of (Murray 2003b, a), in Fig. 10a, and is reduced to a simpler segmented image (Fig. 10c) based on intensity in Fig. 10b. Step function heterogeneities are again used to vary *C* and *a* in simulations shown in Fig. 10d, which demonstrates that spots, labyrinthine, and inverse spot/labyrinthine patterns can all exist together in the same simulation, with boundaries between regions conforming to the underlying heterogeneity.

Finally, we consider a system where we want to preserve a prepattern in some parts of the domain, but undergo a specified Turing-type patterning in other parts of the domain. To proceed, we reconsider the kinetics of Eq. (31), but rather than enforcing the steady state to be $(u,v)=(1,1)$ we consider a steady state at $(u,v)=(U,V)$. We therefore consider the system,

53 $$\begin{aligned}&\frac{\partial u}{\partial t} = \nabla ^2 u+a_1+b_1u^2+c_1u v -C(u-U)^3, \end{aligned}$$

54 $$\begin{aligned}&\frac{\partial v}{\partial t} = D\nabla ^2 v+a_2+b_2v+c_2u v. \end{aligned}$$

Following the same procedure as before (but using conditions around an arbitrary steady state) and setting $F' = S+1$ and $G'=1$, we find

55 $$\begin{aligned}&a_1=V-U,\quad a_2=2V,\quad b_1 =\frac{U-2V}{U}, \quad b_2 = \frac{SU+2U-2V}{V}, \nonumber \\&c_1=U^{-2},\quad c_2=-\frac{S+2}{V}. \end{aligned}$$

Generalising this result so that *U*, or *V*, depends on the spatial variables entails a solution (*U*, *V*) that is no longer a steady solution to the reaction–diffusion system (as the Laplacian term will not vanish), but can instead be thought of as a steady state to the equations in the absence of transport. If the diffusion is sufficiently small, or equivalently the domain is sufficiently large compared to gradients in heterogeneity, then this can be justified asymptotically (Krause et al. 2020b). Asymptotically such a heterogeneous steady state can satisfy only heterogeneous kinetics in the absence of diffusion, but can be driven locally unstable to a more patterned state.

**Fig. 11:**
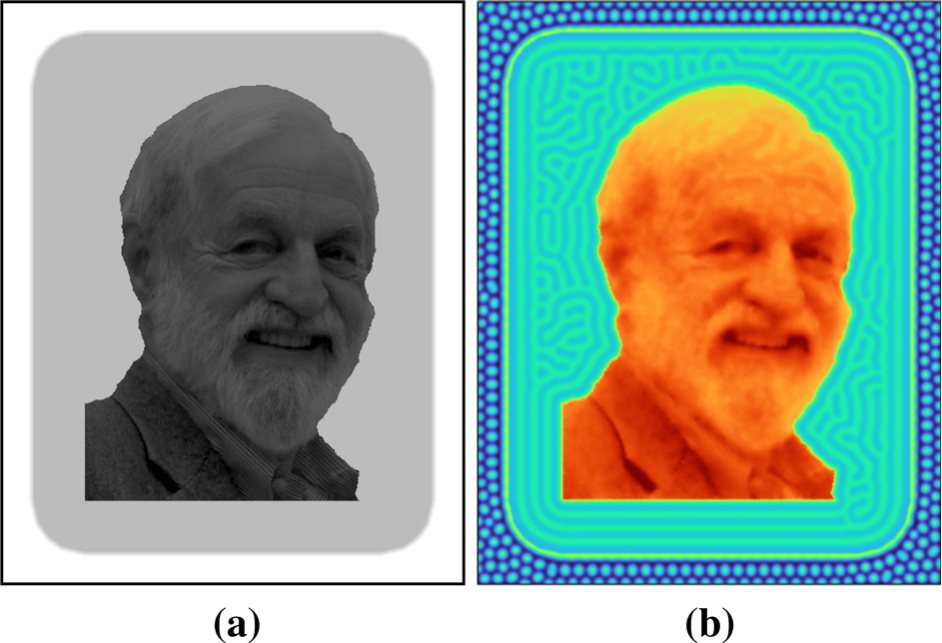
**a** Modified version of Fig. 10**a** to distinguish three regions. The outer background is white, an inner background boundary is light grey, and a central complex inner image is shades of dark grey. **b** Values of *u* from simulations of Eqs. (53)–(55). We take $D=20$ and use a rectangular domain of size 400 by 503. We take a heterogeneous steady state as $(U,V) = (U(x),1)$ and vary the first component in space based on the inverse intensity of the image in (**a**), scaled from 1 in the white region to 11 in the dark region. We take $S=1$ in the outer white and grey regions, and $S=-1$ in the inner region, and $C=0$ in the white region, $C=2$ in the grey region, and $C=20$ in the inner region. In the innermost region, a heterogeneous steady state value is stable to perturbations, and hence, the concentration of *u* can essentially read out the complex spatial heterogeneity

We introduce the heterogeneity similarly to Fig. 10 where distinct regions will have different patterning features, but we want to preserve complex details in the central region of the original image. For this reason, we darken the central region, as shown in Fig. 11a, so that the lightest portion of the central region is darker than the outer part of the image. We also introduce a second grey area around this so that there are three distinct regions: a white outer border, a grey inner border, and darker detailed centre region. In this centre area, we want to stabilise a heterogeneous steady state and so also make *S* depend on space so that the system is only Turing unstable in the outer regions. We will also increase the value of *C* in this region to ensure stripes and labyrinthine patterns in this region. See Fig. 11a for the image used as a heterogeneity, and Fig. 11b for results from our simulation.

By design, Fig. 11b thus shows a transition from spots in the outermost region, to a labyrinthine boundary surrounding a detailed pattern matching the original heterogeneity. Given the size of the domain, and the value of *C* used, the solution in these regions closely mirrors the input pattern, effectively preserving all of the spatial structure inherent in this region. Both modulation of Turing patterns and a morphogen conforming to a complex prepattern directly have been implicated in reconciling Turing-type patterning with other theories of spatial patterning such as positional information (Green and Sharpe 2015). This example shows that matching such predictions can be implemented in a straightforward and systematic manner by suitably defining kinetics without any parameter fitting.

## Discussion

We have presented a relatively straightforward way to construct a rudimentary reaction–diffusion system, which can be designed to have specific simple output patterns, within arbitrarily elaborate parameter regimes. Specifically, we showed how this system can be used to create unbounded, or non-contiguous parameter regimes, as well as how the phase and two-dimensional pattern type can be specified within each region. Such an approach is useful pedagogically and might be employed to design Turing-type systems for use in both applied mathematical research, and plausibly industrial applications, as described in the introduction. Beyond this, our results show reaction–diffusion systems may be designed to match complicated parameter dependencies and simple patterning properties. This suggests that consistency with the patterning of a reaction–diffusion model with freedom in the reaction kinetics may be a relatively weak test of the hypothesis of a Turing patterning mechanism. For instance, when reaction–diffusion systems are evoked to understand heterogeneous biological patterns, the exact kinetic pathways behind a physical application are unknown, since the nature of the morphogens is themselves unspecified. Two populations are presumed to interact through an activator–inhibitor feedback, and the experimental outcome being matched is typically some form of heterogeneous signal, usually taking the form of high and low concentrations as a one-dimensional signal, or as spot, or stripe patterns in two dimensions (Woolley et al. 2014; Cho et al. 2011). Due to this lack of mechanistic detail, there is freedom to suggest any form of kinetics that have the correct interaction form (pure, or cross, kinetics) to produce the final heterogeneous solutions that matches the desired pattern. Usually, these kinetics are taken from a small pool of well understood examples (Gierer and Meinhardt 1972; Schnakenberg 1979; Barrio et al. 1999).

Here, we have simplified the standard Turing conditions down to an inequality on two variables (inequality (17)), assuming that the diffusion ratio and spatial domain can be taken as large as required. This simplification allows us to define a set of cross and pure kinetics that will present a Turing instability in any desired parameter region. The kinetic forms are particularly simple in the case that the parameter region can be specified by level set curves. Finally, we demonstrated that any two-dimensional reaction–diffusion system can be further tailored to produce either a spot or labyrinthine pattern in two dimensions, as required, with mixed patterns also possible when sharp spatial heterogeneities are included in the parameter values.

This suggests that if we are unable to constrain our models, through domain and diffusion scales or kinetic terms, then the reaction–diffusion framework alone is able to produce essentially any simple desired qualitative pattern and is thus automatically consistent with any simple stripe or spot pattern and even patterns with sharp transitions between spots and stripes. Moreover, recent work into larger interaction networks has demonstrated that the Turing instability is even less constrained when more populations are considered. Work by Marcon et al. (2016) shows that there are Jacobian sign structures (dictating local interaction types as activatory, or inhibitory) that are able to pattern without constraining the diffusion ratio parameter, though with the inclusion of immobile species. Further, their analysis demonstrates that Jacobian sign structures specify all the different in and out of phase relationships between the morphogens. Thus, unless detailed knowledge of all species’ interactions is known, there is once again flexibility in the choice of potential kinetics.

Notably, even though the assumptions of large diffusion ratios and large domains are generally necessary, there are many ways these assumptions can be weakened, such as by using substrate binding to slow one of the populations down, creating the large diffusion ratio required for a two species model (Klika et al. 2012). In fact, such mechanisms were exploited in the well-known Lengyel–Epstein system (Lengyel and Epstein 1991), which modelled chemical examples of Turing instabilities. Because of the enormous complexity of biological systems, difficulties with these assumptions are often neglected or presented with a brief mention of including more species (Dougoud et al. 2019; Liu et al. 2006; Cho et al. 2011). Additionally, if theoreticians are able to introduce prepatterns into their simulations then (as seen in Sects. 4 and 4.1), there is scope to combine simpler patterns to generate almost arbitrarily complicated patterns.

Furthermore, modelling certain experimental perturbations is also feasible. Usually, experimental data are focused on generating pattern transitions. Namely, a chemical factor is added into the system, which changes the regulation of an interaction (e.g. upregulation of the influence of the activator on the inhibitor), generating an observable pattern transition between spots to stripes, or, vice versa. Such outcomes can be encompassed in the presented framework. Namely, if we want a pattern to transition between spots and stripes, we can introduce a feedback between the experiment and a cubic term like that seen in Sect. 3.3.

Currently, we have not presented a means of guaranteeing that other mechanisms of patterning will not be feasible outside of $\Omega$, which is equivalent to $S(\varvec{p}) > 0$. Thus, we cannot strictly claim a method of eradicating self-organisation external to $\Omega$ using the kinetic parameters. This will be the subject of future work. However, even without the use of kinetic parameters, if there is freedom to assume experimental perturbations influence diffusion rates, then shrinking the ratio, *D*, will cause patterns to be eradicated, even with parameters such that $S(\varvec{p})>0$.

Nonetheless, such subtleties do not ameliorate the overarching observations that, with free reign, it is feasible to construct kinetics that generate any simple homogeneous, or sharply stratified Turing pattern within any specified region of parameter space. Hence, we conclude that critical consideration is required when reaction–diffusion models are compared with experimental data; especially with regard to the extent, the reaction kinetics have been constrained. In particular, the interpretation of such comparisons is only as informative as the constraints that are placed upon the model, noting that mechanism-based restrictions on the kinetics will reduce such freedoms.

Although the universality of these Turing systems potentially limits the insights that we are able to generate with minimal biological constraints, they are nonetheless useful (Gelfert 2018). For example, recent work with experimentalists on generating patterns with decreasing wavelengths has shown that homogeneous Turing systems are unable to generate sufficient wavelength modulation across a spatial domain. Thus, extra gradients in the kinetics are needed, which then lead to the investigation of whether such gradients exist in the experimental system, as well as an ensuing theory of Turing patterns in heterogeneous domains (Krause et al. 2018b, 2020b).

Equally, experimentalists are constantly working within the freedom of Turing’s theory to try and further constrain possible mechanisms. Recent work by Economou et al. (2020) investigated the biochemical interactions of proteins FGF, Hh, Wnt, and BMP and their ability to generate transverse ridges in the mouths of mice. Critically, by identifying activation patterns and the response of these patterns to multiple inhibitory perturbations they were able to substantially constrain the number of possible kinetics to 154 potential interaction networks. Thus, although we are not quite at the stage where we can uniquely define a set of biological interactions for a given patterning mechanism, we are optimistic that future collaborations could achieve this goal.

Finally, the fundamental idea behind the Turing instability is extremely valuable across the spectrum of experimental, modelling, and theoretical studies. Namely, the Turing instability demonstrates that a system can be more than the sum of its components. A diffusive system is thought to generally dissipate patterns and connecting this to a stable set of kinetics should, intuitively, not create sustained heterogeneity. Thus, philosophically, the Turing pattern demonstrates the need for mathematical modelling of biological ideas, where verbal models and intuition can only take us so far (Maini et al. 2016, 2012; Woolley et al. 2017).

In summary, the richness of pattern exhibited by reaction–diffusion equations and the frequent freedom in the choice of kinetics entails that many putative Turing systems are subject to severe difficulties of model selection and parameter identifiability. This is highlighted here by an explicit and constructive demonstration of how an extremely diverse range of patterns, within any specified region of parameter space, can be reproduced using a non-unique constructive algorithm, as summarised in Fig. 1.

As such pattern matching given freedom in the choice of kinetics is a poor discriminator of mechanism on comparing theoretical and experimental studies. Further understanding the detailed mechanisms of biological self-organisation is required for testing Turing’s hypothesis against experimental studies, and more generally elucidating its place within larger developmental schema, for example. From the experimental side, we would encourage more focus on spatial modulation of patterning features such as wavelength and explicit constraints on signalling networks (e.g. the nonlinear interactions between activator and inhibitor networks). Future, theoretical studies should also address limitations of Turing’s theory, such as the tendency of a limited variation in the wavelength or determining fundamental principles valid beyond two-species models.
